# Preventable causes of cancer in Texas by Race/Ethnicity: Inadequate diet

**DOI:** 10.1016/j.pmedr.2021.101637

**Published:** 2021-11-17

**Authors:** Franciska J. Gudenkauf, Aaron P. Thrift

**Affiliations:** a Section of Epidemiology and Population Sciences, Department of Medicine, Baylor College of Medicine, Houston, TX, USA; b University of Texas Health Science Center at Houston School of Public Health, Houston, TX, USA; c Dan L Duncan Comprehensive Cancer Center, Baylor College of Medicine, Houston, TX, USA

**Keywords:** Population attributable fraction, Cancer disparity, Diet, Fiber intake, Calcium intake, Meat consumption

## Abstract

We estimated the percentage and number of all incident cancer cases diagnosed in Texas in 2015 that were attributable to inadequate diet and examined for racial/ethnic differences. We calculated population attributable fractions for cancers with a causal relationship with red and processed meat consumption, insufficient fiber intake, and insufficient calcium intake, using prevalence estimates from the National Health and Nutrition Examination Survey and relative risk estimates from the World Cancer Research Fund/American Institute for Cancer Research 2018 Third Expert Report. Overall, 3.3% of all new cancers (3,428 cases) diagnosed in Texas in 2015 were attributable to inadequate diet. More diet-associated cancers were diagnosed in men (3.8%) than women (2.9%). Insufficient fiber intake (1.2%) contributed more cancers than processed meat consumption (1.0%), insufficient calcium intake (0.8%), and red meat consumption (0.4%). Non-Hispanic Blacks (4.4%) had a higher proportion of cancers attributable to inadequate diet than Hispanics (3.7%) and non-Hispanic Whites (3.1%). Considering only colorectal cancers, inadequate diet caused 39.6% of cases in non-Hispanic Blacks, compared to 33.6% in non-Hispanic Whites and 33.4% in Hispanics. Inadequate diet serves as an important but preventable source of cancer. In general, and for minority populations specifically, cancer prevention programs should continue to advocate for universal compliance with recommended dietary guidelines.

## 1 Introduction

The World Cancer Research Fund (WCRF)/American Institute for Cancer Research (AICR) 2018 Third Expert Report concluded there is strong evidence (i.e., “convincing” or “probable” protective or causal relationships exist) that aflatoxins, salt preservation, processed meat, red meat, Cantonese-style salted fish, arsenic in drinking water, mate (a caffeinated South American beverage), high-dose beta carotene supplements, and glycemic load cause cancers at different sites, while wholegrains, dietary fiber, non-starchy fruits and vegetables (aggregated), dairy products, milk, cheese, dietary calcium, coffee, and calcium supplements prevent cancer development (World Cancer Research Fund/American Institute for Cancer Research, 2018). Collectively, there are many potential areas for preventative dietary intervention that may help to decrease overall cancer burden, although some may be more relevant than others in the United States. Further, the consumption habits of some dietary exposures vary by race/ethnicity (Storey and Anderson, 2014, Vaccaro and Huffman, 2017), suggesting there may also be racial/ethnic differences in cancers attributable to dietary exposures. Exploring these differences may help to identify the dietary exposures more relevant to cancer burden in certain population subgroups, while advancing cancer prevention.

In this study, we aimed to estimate the population attributable fractions (PAFs) and number of excess cancer cases diagnosed in Texas in 2015 that were attributable to an inadequate diet, which we have defined as not adhering to national or global dietary recommendations. We included in this analysis dietary risk factors for which there is strongest evidence of causality and for which representative prevalence data and risk estimates are available – namely, red meat consumption, processed meat consumption, dietary fiber intake, and dietary calcium intake. WCRF/AICR declared “convincing” evidence that processed meat consumption causes colorectal cancer, while red meat consumption “probably” causes colorectal cancer (World Cancer Research Fund/American Institute for Cancer Research, 2018). Dietary fiber intake (World Cancer Research Fund/American Institute for Cancer Research, 2018) and dietary calcium intake (World Cancer Research Fund/American Institute for Cancer Research, 2018) both “probably” prevent colorectal cancer according to WCRF/AICR. We estimated the PAF for all cancers attributable to inadequate diet, as well as the PAF for colorectal cancers only. We selected Texans as our study population because their rich diversity provides a valuable opportunity to explore racial/ethnic differences in inadequate diet-attributable cancers. By stratifying our analysis according to major racial/ethnic subgroups of the population, we aimed to reveal any racial disparities in the fraction of cancers attributable to each dietary factor and to an inadequate diet overall.

## 2 Methods

The Texas Cancer Registry (TCR) provided counts of new invasive cancer cases diagnosed in Texas in 2015, overall and by age group, sex, and race/ethnicity (Cancer data have been provided by the Texas Cancer Registry). Colorectal cancers were identified in the TCR data file using Surveillance, Epidemiology, and End Results (SEER) Site Recode International Classification of Diseases (ICD)-O-3/World Health Organization (WHO) 2008 Definition codes C18-20 (Recode, 2008).

Relative risk (RR) estimates for associations with red meat (reported per 100 g increase in consumption per day) (World Cancer Research Fund/American Institute for Cancer Research, 2018), processed meat (reported per 50 g increase in consumption per day) (World Cancer Research Fund/American Institute for Cancer Research, 2018), fiber (reported per 10 g increase in intake per day) (World Cancer Research Fund/American Institute for Cancer Research, 2018), and dietary calcium (reported per 200 mg increase in intake per day) (World Cancer Research Fund/American Institute for Cancer Research, 2018) were taken from the WCRF/AICR Third Expert Report. In accordance with previous studies (Nagle et al., 2015, Nagle et al., 2015), we converted RR estimates to estimates of increase in risk per unit consumption or deficit per day (Rg), depending on whether the dietary risk factor is detrimental or protective, respectively. For red and processed meats, the following formula was used to calculate increase in risk per unit consumption per day (Nagle et al., 2015): $\mathrm{Rg}=\frac{ln(RR)}{x}$, where × is the dose–response increment in grams/day reported for that RR (e.g., for processed meat, $\mathrm{Rg}=\frac{ln(RR)}{50}$). For fiber and calcium, the following formula was used to calculate increase in risk per unit deficit per day (Nagle et al., 2015): $\mathrm{Rg}=\frac{\ln\left(\frac{1}{\mathrm{RR}}\right)}{x}$, where × is the dose–response increment in grams/day or milligrams/day reported for that RR (e.g., for calcium intake, $\mathrm{Rg}=\frac{\ln\left(\frac{1}{\mathrm{RR}}\right)}{200}$).

RR and Rg estimates are displayed in Table 1.

**Table 1:** Relative risk measures associated with inadequate diet and colorectal cancer. RR indicates relative risk per incremental increase in consumption per day. Rg indicates increase in risk per unit consumption or deficit per day, depending on whether the dietary risk factor is detrimental or protective.

Dietary Risk Factor	Risk Measure	Men	Women	Persons
Red Meat	RR (per 100 g increase per day)	*1.28*	*1.02*	*1.12*
	Rg (per 1 g increase per day)^a^	0.0025	0.0002	0.0011

Processed Meat	RR (per 50 g increase per day)	*1.11*	*1.18*	1.16
	Rg (per 1 g increase per day)^b^	0.0021	0.0033	0.0030

Fiber	RR (per 10 g increase per day)	0.89	0.91	*0.93*
	Rg (per 1 g deficit per day)^c^	0.0117	0.0094	0.0073

Calcium	RR (per 200 mg increase per day)	0.93	0.93	0.94
	Rg (per 1 mg deficit per day)^d^	0.0004	0.0004	0.0003

*Italics:* reported confidence interval includes null value.

a Rg = ln(RR)/100.

b Rg = ln(RR)/50.

c Rg = ln(1/RR)/10.

d Rg = ln(1/RR)/200.

In the absence of state-based estimates, we used national weighted (to account for oversampling, non-response, and post-stratification) (Health and Examination, 2019) prevalence estimates from the National Health and Nutrition Examination Survey (NHANES) Dietary Data as proxies for Texas (CDC, 2005, CDC, 2009). We used the same single source (NHANES) for all population sub-groups examined in this study. We assumed a latency period of ten years between exposure and outcome (i.e., inadequate diet and new cancer diagnosis), similar to previous studies (Nagle et al., 2015, Nagle et al., 2015). Hence, we used NHANES Dietary Data from survey years 2005–2006 when available (for fiber and calcium). For red meat and processed meat, the 2005–2006 survey questions were not specific toward red meat and processed meat, so consumption could not be accurately quantified. However, the 2009–2010 survey quantified specific red meat consumption and processed meat consumption separately, and neither red meat consumption nor processed meat consumption has changed significantly between 1999 and 2012 (Rehm et al., 2016). Thus, we applied the 2009–2010 prevalence data for meat consumption as a surrogate for 2005–2006 prevalence estimates.

Recommended consumption levels were derived from the U.S. Department of Health and Human Services’ and the U.S. Department of Agriculture’s *2015*–*2020 Dietary Guidelines for Americans* when available (U.S. Department of Health and Human Services and U.S. Department of Agriculture, 2015). For meat consumption (red and processed), these guidelines did not provide specific recommendations, instead grouping together meats, poultry, and eggs (U.S. Department of Health and Human Services and U.S. Department of Agriculture, 2015), so we used WCRF/AICR’s Third Expert Report instead (World Cancer Research Fund/American Institute for Cancer Research, 2018). Consumption above recommended levels was considered detrimental for red and processed meat. Although the Third Expert Report specifically discusses the protective effects of fiber and calcium on colorectal cancer risk (World Cancer Research Fund/American Institute for Cancer Research, 2018, World Cancer Research Fund/American Institute for Cancer Research, 2018), we focus instead on cancers attributable to insufficient intake of these dietary elements; thus, fiber and calcium intake below recommended levels was considered insufficient. Prevalence data were categorized according to consumption levels defined in Supplementary Table 1. Prevalence estimates for consumption of red meat, processed meat, fiber, and calcium were stratified by age group, sex, and race/ethnicity. Table 2 shows the prevalence of adults not adhering to dietary recommendations.

**Table 2:** Prevalence of Americans aged ≥ 18 years not meeting recommendations for red meat, processed meat, fiber, and calcium consumption (%), overall and by race/ethnicity.

		Men				Women				Persons			
		Red Meat^a^	Processed Meat^a^	Fiber	Calcium	Red Meat^a^	Processed Meat^a^	Fiber	Calcium	Red Meat^a^	Processed Meat^a^	Fiber	Calcium
All		59.9	88.1	88.0	53.4	45.4	83.9	95.6	71.7	52.7	86.0	92.0	63.0
Race/Ethnicity													
	Non-Hispanic Whites	63.9	91.6	87.8	49.5	48.7	85.9	95.6	69.3	56.4	88.8	91.9	59.9
	Non-Hispanic Blacks	49.0	86.6	96.4	67.4	37.6	86.7	98.3	86.6	42.9	86.6	97.5	78.2
	Hispanics	52.8	84.9	79.9	63.6	40.8	79.8	92.9	69.0	47.3	82.5	86.6	66.4
	Other Races/Ethnicities	53.8	62.5	89.3	57.2	36.6	67.9	94.0	74.6	44.9	65.3	91.7	66.2

Red meat recommended consumption: ≤60 g/day.

Processed meat recommended consumption: 0 g/day.

Fiber recommended intake: ≥28 g/day.

Calcium recommended intake: ≥1000 mg/day.

Note: totals may not sum manually due to rounding.

a Prevalence data sourced from NHANES 2009–2010 given evidence that consumption has remained stable since 2006. Prevalence data only available for adults aged 18–69.

For each dietary exposure, we used standard formulae to calculate PAFs by sex, age group, and race/ethnicity: $\mathrm{PAF}=\frac{\sum (p_{x}*{\mathrm{ERR}}_{x})}{1+\sum (p_{x}*{\mathrm{ERR}}_{x})}$, where p_x_ is the population proportion at consumption level × (x = 1,…4 or 5 according to the dietary consumption levels as defined in Supplementary Table 1), and the excess relative risk ${\mathrm{ERR}}_{x}=\left(e^{\left(Rg\times G_{x}\right)}-1\right)$, where Rg is as defined above and G_x_ is the average excess or deficit consumption within each exposure category × (in grams/day or milligrams/day) (Nagle et al., 2015). For detrimental exposures, G_x_ = (median consumption within exposure category x) – (reference level for exposure), and for protective exposures, G_x_ = (reference level for exposure) – (median consumption within exposure category x). Calculated PAFs were then multiplied by incident colorectal cancer counts to estimate the number of excess colorectal cancer cases attributable to each dietary exposure, by sex, age group, and race/ethnicity. To accommodate the latency period, prevalence data and corresponding PAFs in each age group were paired with the cancer incidence age group 10 years older (e.g., 2005–2006 prevalence data for age group 25–34 years was paired with 2015 cancer counts for age group 35–44 years). Total excess colorectal cancer cases as a fraction of all incident cancers represented the percentage of all incident cancers (excluding basal cell carcinoma [BCC] and squamous cell carcinoma [SCC] of the skin) that were attributable to each dietary exposure. Finally, we added all excess colorectal cancers attributable to all four dietary exposures to calculate the percentage of colorectal cancers and all incident cancers diagnosed in Texans aged ≥ 25 years in 2015 that were attributable to inadequate diet.

## 3 Results

NHANES data show that the majority of Americans (including Texans) aged ≥ 18 years in 2006 did not adhere to nutritional recommendations for red meat (52.7%), processed meat (86.0%), fiber (92.0%), and calcium (63.0%). More men than women did not adhere to guidelines for the consumption of red and processed meat, while women were more likely than men to not adhere to fiber and calcium intake recommendations (Table 2). Non-Hispanic Whites were more likely than the other racial/ethnic groups to consume excess red and processed meats. However, the prevalence of insufficient fiber and calcium intake was highest in non-Hispanic Blacks.

In Texan adults aged ≥ 25 years in 2015, there were 103,408 cancer cases diagnosed (excluding BCC and SCC of the skin), with 51,472 cases in men and 51,936 cases in women. The majority of cases were diagnosed in non-Hispanic Whites (65,214 cases), followed by Hispanics (22,642 cases) and non-Hispanic Blacks (12,020 cases); the remaining 3,532 cases occurred in individuals of Other Races/Ethnicities. Overall, 3.3% of all new cancer cases or 3,428 excess cancers (excluding BCC and SCC of the skin) diagnosed in Texas in 2015 were attributable to inadequate diet (Table 3). Men (3.8%, 1,935 excess cases) had a numerically higher proportion of cancers attributable to inadequate diet than women (2.9%, 1,493 excess cases). Of the dietary factors analyzed, insufficient fiber intake had the highest PAF at 1.2% or 1,236 excess cases (processed meat: 1.0%, 1,002 excess cases; calcium: 0.8%, 811 excess cases; red meat: 0.4%, 379 excess cases). Men had numerically higher overall dietary factor-specific PAFs than women for red meat consumption (men, 0.7%; women, 0.0%) and insufficient fiber intake (men, 1.3%; women, 1.0%).

**Table 3:** Age-weighted PAFs of cancers attributable to inadequate diet in Texas in 2015 by race/ethnicity (%), adults aged ≥ 25 years.

	Red Meat		Processed Meat		Insufficient Fiber		Insufficient Calcium		All Dietary Factors	
	Colorectum	All cancers*	Colorectum	All cancers*	Colorectum	All cancers*	Colorectum	All cancers*	Colorectum	All cancers*
**ALL**										**PAF** (excess cases)
**Men**	6.5	**0.7** (3 6 5)	9.0	**1.0** (5 0 5)	12.3	**1.3** (6 9 5)	6.6	**0.7** (3 7 0)	34.4	**3.8** (1935)
**Women**	0.3	**0.0** (14)	11.1	**1.0** (4 9 7)	12.1	**1.0** (5 4 1)	9.9	**0.8** (4 4 1)	33.5	**2.9** (1493)
**Persons**	3.8	**0.4** (3 7 9)	9.9	**1.0** (1002)	12.3	**1.2** (1236)	8.0	**0.8** (8 1 1)	34.0	**3.3** (3428)

**NON-HISPANIC WHITES**										**PAF** (excess cases)
**Men**	6.8	**0.7** (2 2 3)	9.4	**0.9** (3 0 8)	12.1	**1.2** (3 9 3)	5.8	**0.6** (1 9 0)	34.2	**3.4** (1114)
**Women**	0.3	**0.0** (9)	11.3	**0.9** (3 0 0)	12.0	**1.0** (3 1 7)	9.3	**0.8** (2 4 8)	32.9	**2.7** (8 7 4)
**Persons**	3.9	**0.4** (2 3 2)	10.3	**0.9** (6 0 8)	12.0	**1.1** (7 1 1)	7.4	**0.7** (4 3 8)	33.6	**3.1** (1989)

**NON-HISPANIC BLACKS**										**PAF** (excess cases)
**Men**	5.8	**0.7** (41)	9.5	**1.1** (68)	14.9	**1.8** (1 0 6)	9.5	**1.1** (68)	39.7	**4.7** (2 8 3)
**Women**	0.2	**0.0** (1)	12.7	**1.3** (79)	13.8	**1.4** (86)	12.9	**1.3** (81)	39.6	**4.1** (2 4 8)
**Persons**	3.2	**0.4** (43)	11.0	**1.2** (1 4 7)	14.3	**1.6** (1 9 2)	11.1	**1.2** (1 4 9)	39.6	**4.4** (5 3 1)

**HISPANICS**										**PAF** (excess cases)
**Men**	5.2	**0.7** (78)	7.4	**1.0** (1 1 2)	11.5	**1.6** (1 7 3)	9.8	**1.4** (1 4 8)	34.0	**4.8** (5 1 1)
**Women**	0.3	**0.0** (3)	10.4	**0.9** (1 0 7)	11.6	**1.0** (1 1 9)	10.2	**0.9** (1 0 5)	32.5	**2.8** (3 3 4)
**Persons**	3.2	**0.4** (81)	8.7	**1.0** (2 1 9)	11.6	**1.2** (2 9 2)	10.0	**1.1** (2 5 3)	33.4	**3.7** (8 4 5)

**OTHER RACES/ETHNICITIES**										**PAF** (excess cases)
**Men**	5.3	0.5 (8)	4.0	0.4 (6)	12.4	1.1 (18)	7.2	0.6 (11)	28.9	**2.5** (43)
**Women**	0.1	0.0 (0)	6.6	0.5 (10)	11.4	1.0 (17)	10.9	0.9 (17)	28.9	**2.4** (44)
**Persons**	2.6	**0.2** (8)	5.3	**0.5** (16)	11.9	**1.0** (36)	9.1	**0.8** (27)	28.9	**2.5** (87)

*Excluding basal cell carcinoma and squamous cell carcinoma of the skin. All cancers combined are displayed as PAF (excess cases).

Note: totals may not sum manually due to Microsoft Excel rounding.

Racial/ethnic subgroup analysis revealed that 4.4% of all cancers (531 excess cases) in non-Hispanic Blacks were attributable to inadequate diet, as compared to 3.7% (845 excess cases) in Hispanics and 3.1% (1,989 excess cases) in non-Hispanic Whites. Non-Hispanic Blacks also had numerically higher overall cancers attributable to processed meat consumption (non-Hispanic Blacks, 1.2%; Hispanics, 1.0%; non-Hispanic Whites, 0.9%), insufficient fiber intake (non-Hispanic Blacks, 1.6%; Hispanics, 1.2%; non-Hispanic Whites, 1.1%), and insufficient calcium intake (non-Hispanic Blacks, 1.2%; Hispanics, 1.1%; non-Hispanic Whites, 0.7%) than the other racial/ethnic subgroups. For red meat consumption, there was no difference in overall PAFs (0.4%) across racial/ethnics subgroups. Within all racial/ethnic subgroups, men had a numerically higher overall PAF for all dietary factors combined than women, although this difference was most pronounced in Hispanics (Table 3).

Considering only colorectal cancers diagnosed in 2015, inadequate diet caused 34.0% of cases. In men, 34.4% of colorectal cancers were attributable to inadequate diet, while in women, this estimate was 33.5%. Almost 40% of colorectal cancers were caused by inadequate diet in non-Hispanic Blacks, compared to 33.6% in non-Hispanic Whites and 33.4% in Hispanics. Insufficient fiber consumption caused 12.3% of colorectal cancers, and 9.9% of colorectal cancers were attributable to processed meat consumption. The PAF for colorectal cancer due to insufficient calcium intake was 8.0%, while for red meat consumption, it was 3.8%.

## 4 Discussion

Our analysis estimated 3.3% of all incident cancer cases (3,428 excess cancers, excluding BCC and SCC of the skin) or 34.0% of colorectal cancer cases diagnosed in Texas in 2015 in adults aged ≥ 25 years were attributable to inadequate diet, with more cases in men (3.8%) than women (2.9%). Non-Hispanic Blacks (4.4%) had a numerically higher proportion of all cancers attributable to inadequate diet than Hispanics (3.7%) and non-Hispanic Whites (3.1%). Insufficient fiber consumption (1.2%) contributed the highest proportion of all cancer cases compared to processed meat consumption (1.0%), insufficient calcium intake (0.8%), and red meat consumption (0.4%).

Our findings add to the increasing collection of research showcasing the significance of dietary risk factors as a source of cancer incidence. A prior U.S.-wide PAF study estimated that 0.9% of cancers (excluding nonmelanoma skin cancers) in 2014 in Americans aged ≥ 30 years were attributable to low fiber, 0.8% to processed meat, 0.5% to red meat, and 0.4% to low calcium (Islami et al., 2018). These results are comparable to ours, although our overall PAFs are slightly higher than in the U.S.-wide study, except for red meat consumption. Further, we found that insufficient calcium intake contributed more to cancer burden in Texas than red meat consumption, which was unlike the U.S.-wide study. However, the U.S.-wide study used 9 g/day as their reference level for red meat consumption (Islami et al., 2018), whereas we allowed up to 60 g/day, per WCRF’s recommendations (World Cancer Research Fund/American Institute for Cancer Research, 2018). This may explain why a slightly higher PAF was estimated for red meat consumption in the U.S. Results from the U.S.-wide study also showed PAFs for colorectal cancer of 10.3% for low fiber (Texas, 12.3%), 8.2% for processed meat (Texas, 9.9%), 5.4% for red meat (Texas, 3.8%), and 4.9% for low calcium (Texas, 8.0%) (Islami et al., 2018). Variation may be partially explained by differences in methodology, such as differences in reference levels and years analyzed for prevalence data. Yet, despite differences in PAF magnitudes, the U.S.-wide study and our study both identified insufficient fiber intake as the most significant contributor to overall cancer burden (all persons) among the other dietary risk factors considered in this analysis, which is likely due to the extremely high prevalence of adults not meeting fiber intake recommendations.

Compared to other countries where similar PAF studies have been conducted, our calculated PAFs are generally lower. In Australia in 2010, 2.3% of all cancers were attributable to deficit in fiber intake (Nagle et al., 2015) (compared to 1.2% in Texas), and 2.3% of cancers were attributable to meat consumption (Nagle et al., 2015) (red and processed combined; compared to 1.3% in Texas, data not shown). Colorectal cancer-specific PAFs were 17.6% for deficit in fiber intake (Nagle et al., 2015) (compared to 12.3% in Texas) and 17.7% for meat consumption (Nagle et al., 2015) (red and processed combined; compared to 13.7% in Texas, data not shown). Meanwhile, in the United Kingdom in 2010, PAFs were 1.5% for all cancers and 12.2% for colorectal cancers caused by deficit in fiber consumption (Parkin and Boyd, 2011), and 2.7% for all cancers and 21.1% for colorectal cancers caused by meat consumption (red and processed combined) (Parkin, 2011). However, national dietary recommendations differ across countries, so direct comparison is difficult.

We found that inadequate diet explained a numerically higher proportion of cancer burden for men than women. The greatest difference in PAF was for cancers caused by excess red meat consumption. This observation is generally consistent with findings from other studies, although there was some variation across studies when considering specific dietary factors by sex. None of the prior PAF studies have evaluated the role of race/ethnicity as an effect modifier of cancers attributable to inadequate diet. In our analysis, we found that non-Hispanic Blacks (4.4%) had a numerically higher overall PAF for all dietary factors combined than Hispanics (3.7%) and non-Hispanic Whites (3.1%). Further, non-Hispanic Blacks had higher overall PAFs for processed meat consumption, insufficient fiber intake, and insufficient calcium intake. The finding that PAFs for insufficient fiber and calcium intake were highest in non-Hispanic Blacks reflects the higher prevalence of non-Hispanic Blacks with insufficient fiber and calcium intake as compared to other racial/ethnic subgroups.

Several of the dietary risk factors excluded from this study are worth mentioning. Although prevalence data and risk estimates were available for consumption of fruits and vegetables, WCRF/AICR’s conclusion referred only to aggregated fruits and vegetables “probably” preventing aggregated aerodigestive cancers (World Cancer Research Fund/American Institute for Cancer Research, 2018); however, there was only “limited” evidence for a protective effect when fruits and vegetables were considered separately or cancer sites were considered separately (World Cancer Research Fund/American Institute for Cancer Research, 2018). Further, the International Agency for Research on Cancer (IARC), which is charged with designating exposures as cancer-causative or cancer-preventative, also declared in 2003 that there was limited evidence for a protective relationship between fruit and vegetable consumption and certain cancers (IARC, 2003). Hence, we deemed the evidence to be lacking and so excluded fruits and vegetables from this analysis. Dairy products, milk, and cheese were excluded, not only because prevalence data were incomprehensive, but also because the protective nature of dairy products on cancer development is largely attributable to calcium content, which we included in this study (World Cancer Research Fund/American Institute for Cancer Research, 2018, Islami et al., 2018). For supplements, although prevalence data were available, ideal risk estimates were not, and WCRF/AICR maintains that supplements should not be used for cancer prevention, despite the protective effect of calcium supplements on colorectal cancer risk (World Cancer Research Fund/American Institute for Cancer Research, 2018); thus, supplements were excluded from our study.

Our study has some limitations. We made several assumptions within this study: that national NHANES prevalence data were representative of Texas, although there may be differences in population prevalence due to differences in demographics; that the latency period between dietary risk factor exposure and cancer development is ten years, although latency periods are unknown (Nagle et al., 2015, Nagle et al., 2015), and dietary exposure likely has a cumulative effect on cancer risk; that NHANES 2009–2010 prevalence data for red and processed meat consumption is representative of 2005–2006 meat consumption rates, given a history of stable prevalence (Rehm et al., 2016); and that average reference levels, which we derived from national and global dietary guidelines, apply to the entire population, although guidelines differ by sex and age group. If, for example, red meat consumption is higher in Texas than America overall, we may have under-estimated the PAFs for red meat. For fruit and vegetable consumption, the percent of Texans (BRFSS, 2021) who ate fruits and vegetables more than 5 times a day was not different from Americans overall (both approximately 23%) (CDC, 2005, CDC, 2009). Further, while we may have under/over-estimated the PAFs if the U.S.-based estimates are not representative of Texas, the internal comparisons between racial/ethnic subgroups remain valid because dietary consumption estimates were obtained from a single source (i.e., all races/ethnicities are equally impacted by potential Texas vs. U.S.-overall differences). Additionally, our selection of reference levels based on dietary guidelines may not be consistent with the biological point that confers an increased risk of cancer; for example, we used 28 g/day of fiber consumption as our reference level, although consumption of less than 28 g/day may not necessarily correlate with the biological point at which insufficient fiber consumption increases cancer risk. Also, prevalence estimates from NHANES were based on self-reported 24-hour dietary recall, which may introduce reporting bias, as dietary recall is inherently subjective, inconsistent, and difficult. Further, NHANES data for meat consumption were only available until age 69, so we applied prevalence estimates from ages 60–69 years to the age group ≥ 70 years; however, this may not have been representative of actual consumption prevalence in this age group. Additionally, diet is deeply intertwined with other cancer risk factors, like alcohol, obesity and physical inactivity. Although the numbers of cancers attributable to inadequate diet generated by these analyses appear precise, we remind readers that there is potential for error in these estimates due both to statistical uncertainty (precision) as well as variation in prevalence and risk estimates. We did not calculate confidence intervals for the PAF as there is no universally agreed approach. Although we used adjusted RR estimates from large prospective cohort studies or *meta*-analyses of prospective studies, the RRs from these studies may be affected by residual confounded by unmeasured or poorly measured variables. As we did not remove individuals with confounding factors from our analysis, similar to other population-based studies (World Cancer Research Fund/American Institute for Cancer Research, 2018, World Cancer Research Fund/American Institute for Cancer Research, 2018, Nagle et al., 2015, Nagle et al., 2015), our PAF estimates may similarly be confounded. As this is a descriptive study, any differences reported between subgroups are purely numerical as opposed to statistically significant; thus, findings should be interpreted with caution. Lastly, the Other Races/Ethnicities category is not meant to represent other named races/ethnicities that do not identify as non-Hispanic Black, non-Hispanic White, or Hispanic, but merely serves as a category for individuals who do not identify with these major population racial/ethnic subgroups.

In summary, we estimated that 3.3% of all new cancers (3,428 excess cancers) diagnosed in Texas in 2015 were attributable to inadequate diet. This corresponded to 34.0% of new colorectal cancers. More cancer cases were attributable to inadequate diet in men (3.8%) than women (2.9%), and in non-Hispanic Blacks (4.4%) than Hispanics (3.7%) and non-Hispanic Whites (3.1%). Thus, dietary intervention should continue to be a major priority for cancer prevention programs. Importantly, differences in the proportions of cancers attributable to inadequate diet across racial/ethnic subgroups should be recognized in order to appropriately direct cancer prevention efforts and reduce cancer health inequities.

## Funding

Funds for this study came from Baylor College of Medicine to the laboratory of Aaron P. Thrift.

## Declaration of Competing Interest

The authors declare that they have no known competing financial interests or personal relationships that could have appeared to influence the work reported in this paper.
