# Supplementary material for: Preventable causes of cancer in Texas by Race/Ethnicity: Inadequate diet
Source: Prev Med Rep. 2021 Nov 17;24:101637. doi: 10.1016/j.pmedr.2021.101637 (PMC8684018; doi:10.1016/j.pmedr.2021.101637)
Supplement: Supplementary data 1 [file mmc1.docx]

**Supplementary Table 1.** Recommended consumption levels and categories and sources of prevalence data by dietary factor.

| **Dietary Factor** | **Red Meat** | **Processed Meat** | **Fiber** | **Calcium** |
| --- | --- | --- | --- | --- |
| **Recommended level (per 2,000 kcal diet)** | ≤60 grams/day | 0 grams/day | ≥28 grams/day | ≥1000 milligrams/day |
| **Source of recommendation** | WCRF/AICR’s Third Expert Report:^15^ ≤3 portions/week ≈ 350-500 grams/week ≈ 12-18 ounces/week cooked red meat.  We used the midpoint of this range, i.e., 425 grams/week ≈ 60 grams/day. | WCRF/AICR’s Third Expert Report:^15^ “very little, if any.” | U.S. Department of Health and Human Services’ and the U.S. Department of Agriculture’s *2015-2020 Dietary Guidelines for Americans*:^14^ 14 grams/ day per 1,000 kcal. | U.S. Department of Health and Human Services’ and the U.S. Department of Agriculture’s *2015-2020 Dietary Guidelines for Americans*:^14^ ≥1000 milligrams/day.  *Women ≥51 years and men ≥71 years should consume 1,200 milligrams/day, but we chose a reference level of 1,000 milligrams/day for consistency |
| **Categories for analysis** | ≤60 grams/day (referent group; those adhering to recommended level)  60-80  80-100  100-120  120-140  >140 (group with greatest excess consumption, nearly 1 portion/day) | 0 grams/day (referent group; those adhering to recommended level)  0-35  35-70  70-105  105-140  >140 (group with greatest excess consumption, nearly 1 portion/day) | ≥28 grams/day (referent group; those adhering to recommended level)  21-28  14-21  7-14  0-7 (group with the greatest deficit from referent group, or highest level of insufficient intake) | ≥1000 milligrams/day (referent group; those adhering to recommended level)  800-1000  600-800  400-600  200-400  0-200 (group with the greatest deficit from referent group, or highest level of insufficient intake) |
| **Source of prevalence data** | 2009-2010 NHANES Dietary Screener Questionnaire:^12^ consumption reported by frequency (e.g., 1 time/week). We assumed 1 time/week equates to 1 portion/week and converted consumption to grams/day.  *NHANES only provided prevalence data for meat consumption until age 69 years. Thus, we estimated prevalence for ages ≥70 years from the prevalence for ages 60-69 years. | | 2005-2006 NHANES Dietary Interview – Total Nutrient Intakes:^11^ intake reported in grams for two 24-hour dietary recall interviews (3-10 days apart). We averaged intake from both interviews for each respondent. | |
